# Probing spermiogenesis: a digital strategy for mouse acrosome classification

**DOI:** 10.1038/s41598-017-03867-7

**Published:** 2017-06-16

**Authors:** Alessandro Taloni, Francesc Font-Clos, Luca Guidetti, Simone Milan, Miriam Ascagni, Chiara Vasco, Maria Enrica Pasini, Maria Rosa Gioria, Emilio Ciusani, Stefano Zapperi, Caterina A. M. La Porta

**Affiliations:** 1 Center for Complexity and Biosystems University of Milano, via Celoria 16, 20133 Milano, Italy; 2 Department of Physics, University of Milano, Via Celoria 16, 20133 Milano, Italy; 3 CNR-Consiglio Nazionale delle Ricerche, ISC, Via dei Taurini 19, 00185 Roma, Italy; 4 ISI Foundation, Via Chisola 5, 10126 Torino, Italy; 5 Department of Environmental Science and Policy, University of Milano, via Celoria 26, 20133 Milano, Italy; 6 Department of Biosciences University of Milano, via Celoria 26, 20133 Milano, Italy; 7 Istituto Neurologico Carlo Besta, Via Celoria, 11, 20133 Milano, Italy; 8 Department of Applied Physics, Aalto University, P.O. Box 11100, FIN-00076 Aalto Espoo, Finland; 9 CNR-Consiglio Nazionale delle Ricerche, ICMATE, Via Roberto Cozzi 53, 20125 Milano, Italy

## Abstract

Classification of morphological features in biological samples is usually performed by a trained eye but the increasing amount of available digital images calls for semi-automatic classification techniques. Here we explore this possibility in the context of acrosome morphological analysis during spermiogenesis. Our method combines feature extraction from three dimensional reconstruction of confocal images with principal component analysis and machine learning. The method could be particularly useful in cases where the amount of data does not allow for a direct inspection by trained eye.

## Introduction

Spermatogenesis is a dynamic process during which undifferentiated diploid stem cells mature to differentiated haploid cells called spermatozoa. Mammalian spermatogenesis occurs within the seminiferous tubules and consists of three phases: a mitotic phase in which spermatogonia divide mitotically; a meiotic phase in which spermatocytes divide to form haploid round spermatids and a third phase, called spermiogenesis, in which spermatids encompass morphological changes including acrosome formation, chromatin condensation, and flagellum development resulting in the formation of spermatozoa^1–7^.

A key element of spermiogenesis is the mammalian sperm acrosome, an exocytotic vesicle present on the apical surface of the head^8, 9^ whose correct formation is crucial for the successful fertilization of the egg^10^. Acrosomal biogenesis takes place at the initial step of spermiogenesis and can be divided into four phases that cumulatively complete in about 2 weeks in the mouse and in 1 month in the humans^8–15^. In rodent spermatids, proacrosomal vesicles (granules) containing a variety of proteins assemble and fuse to form a single sphere acrosomal granule in the center of the acrosomal vesicle at the Golgi phase. At the cap phase, the acrosomal granule forms a head cap-like structure that gradually enlarges to cover the nucleus. The head cap continues to elongate outlining the dorsal edge, protruding apically at the acrosome phase, and finally the structure of the acrosome is completed at the end of maturation phase^12^.

Our current understanding of human reproduction is increasing thanks to the use of Assisted Reproductive Techniques (ART) and many studies aim to find a better way to select viable sperm^16^. Even though many aspects of sperm formation have been investigated, only few studies report quantitative measurements of sperm and its components, mainly focusing on the whole sperm heads^17, 18^. Since infertility is a common problem for men, it would be useful to devise standard parameters that could help in ART. A correct formation of the acrosome is crucial for a physiological reproduction capability and the quantification of the ratio between spermatides and spermatozoa can be a valid support for the correct prognosis of diseases linked to an impaired biogenesis of sperm cells.

Conventional strategies to study mammal spermiogenesis usually try to characterize specific morphological features supposed to play a key role in the development of the cells to spermatozoa with the aim of targeting them for possible prognostic/therapeutic strategies. The morphological analysis of spermatozoa is usually performed by a trained eye, but due to the increasing amount of digital images stored, it is becoming important to develop automatic techniques of classification and diagnosis. In this respect, there is still a pressing need to develop reliable automated method for cell morphology assessment. While objective tools for sperm motility assessment exist^19^, current automatic methods for sperm morphology are still not accurate and difficult to use^20^. Hence, subjective morphology sperm cell assessment is the standard in laboratories but results in large variability in the outcome. Machine learning-based intelligent systems could play a pivotal role to reach this goal. The method starts from an input feature matrix, including characteristic values of designated positive and negative samples, and self-trains the prediction models by learning the patterns in the feature matrix. The final goal is then to be able to automatically classify a data set with unknown labels.

In this paper, we present a machine learning approach to classify in a quantitative and semi-automatic way important morphometric characteristics of mammalian acrosomes during spermatogenesis. We start by a three-dimensional digital reconstruction of confocal images of acrosomes from which we extract a discretized mesh representing the surface of each acrosome. We then compute a series of morphological parameters such as volume, surface and local curvatures. These morphological parameters represent the features that will then be analyzed through machine learning and principal component analysis. We illustrate the method by analyzing acrosomes from spermatides and spermatozoa, obtained from seminiferous tubules of young mice, which are known to have different shapes. The ground truth is established by direct classification by eye and the results compared with automatic methods based on machine learning.

## Results and Discussion

**Figure 1:**
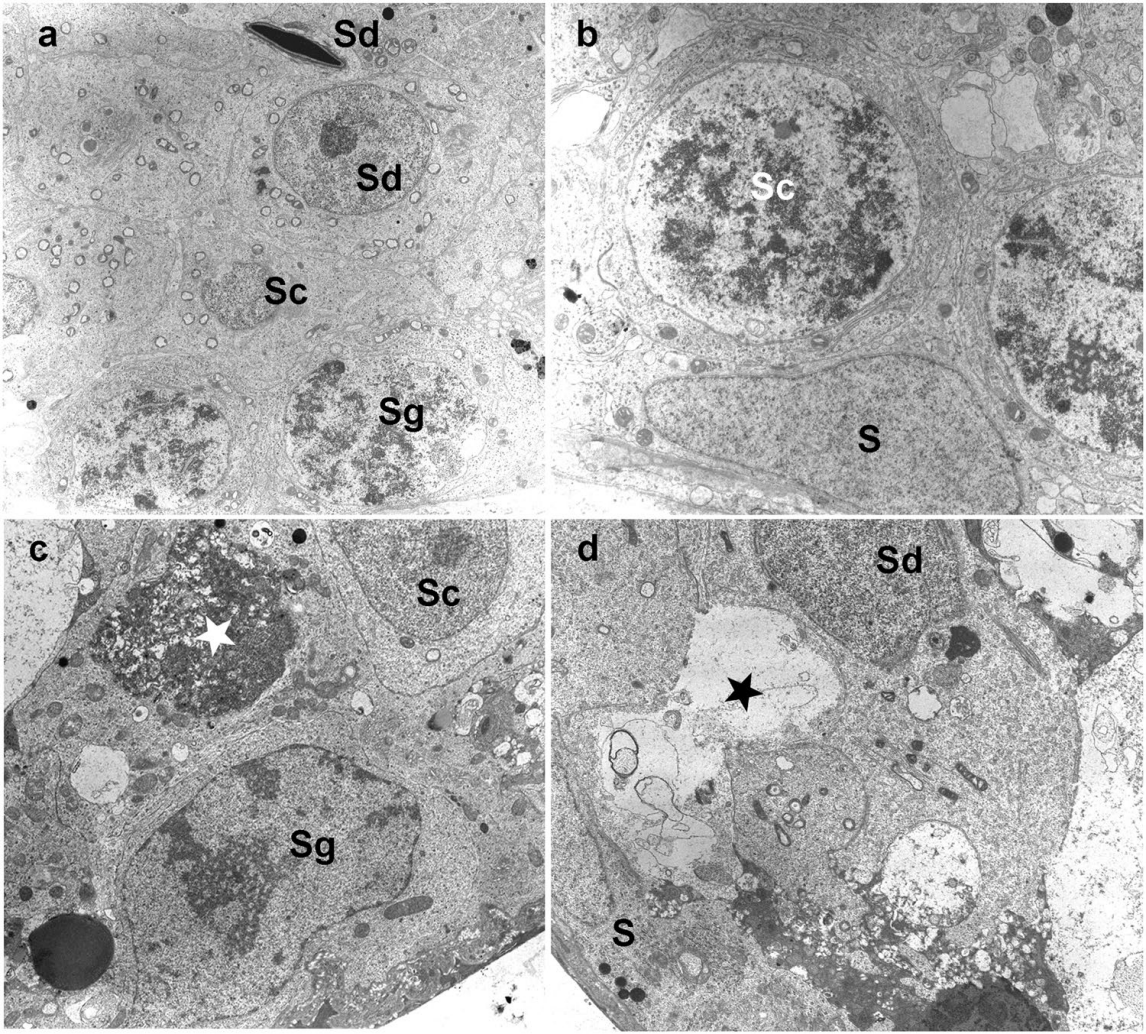
Transmission electron micrograph of mouse seminiferous epithelium. Adult testis tubules obtained as described in Materials and Methods section were immediately fixed (time T0) or after 1 day in culture (time T1). (**a**,**b**) At T0 a well preserved tubular basal compartment of a stage VII tubule shows normal Sertoli cells (S), spermatogonia (Sg), primary spermatocytes (Sc) and spermatids (Sd). x 3500–4800. (**c**,**d**) At T1 the tubular basal compartment shows some signs of cellular degeneration (*). x 4800.

Here we develop a new method combining computational science, quantitative biology and machine learning to classify acrosomes, distinguishing spermatides from spermatozoa in a semi-automatic way, obtaining robust quantitative morphological observables. To this end, we carry out a 3D reconstruction of the surface of acrosomes of spermatides and spermatozoa from sexually mature healthy mice maintained *in vitro* for a few days. Quantifying differences in the fraction of spermatides and spermatozoa could be useful to detect in advance important pathological conditions related to sterility and have impact of ART^17, 18^. In order to maximize the number of acrosomes for the analysis, we carried out the 3D reconstruction of the acrosomes in cells extracted from seminiferous tubules and imaged at different times, either immediately (time T0) or maintained *in vitro* overnight (time T1). An analysis by electron microscopy shows that the overall architecture is preserved between T0 and T1 (Fig. 1) and we did not record any statistical difference in the quantitative parameters extracted from confocal images.

**Figure 2:**
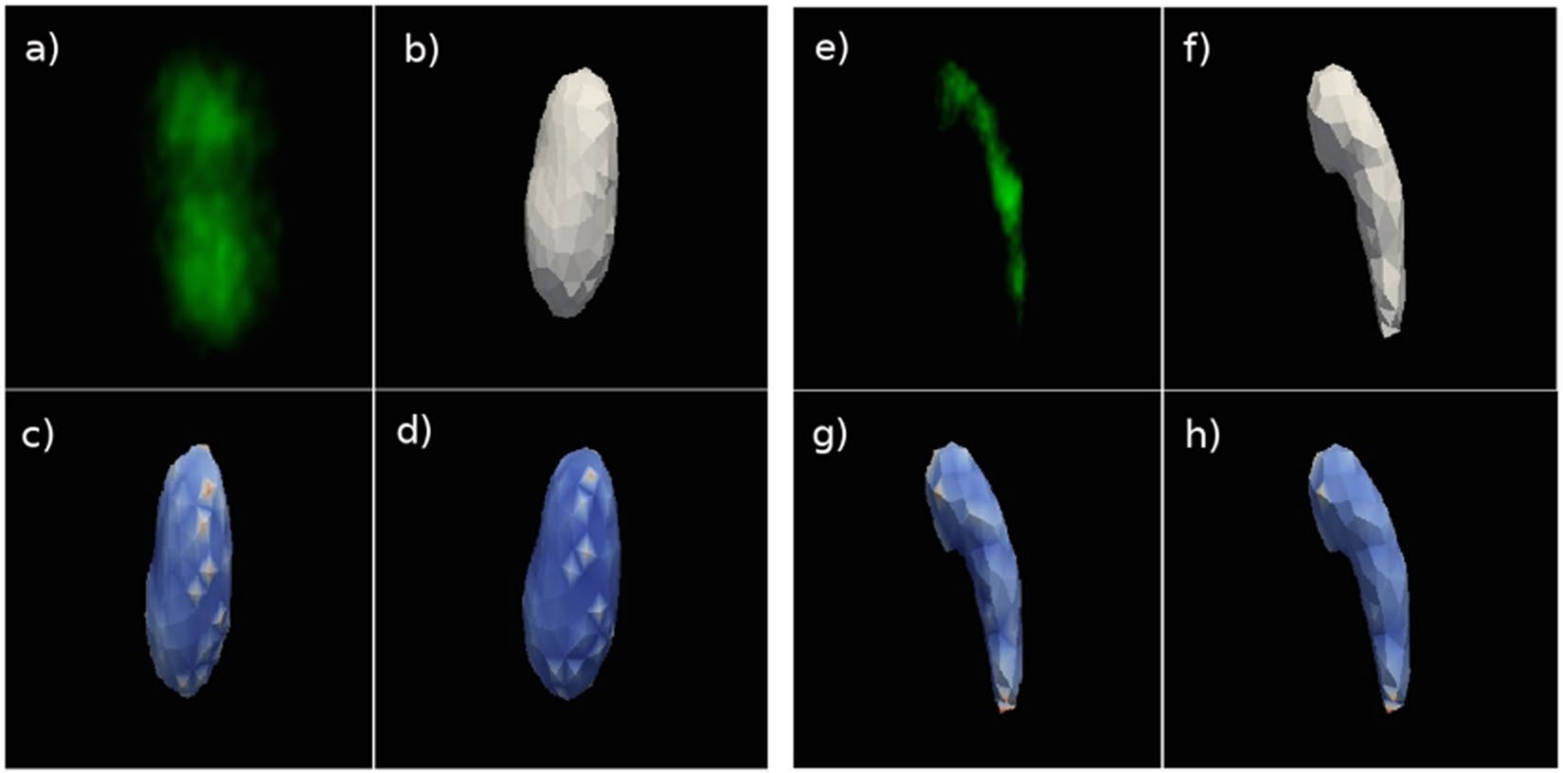
Acrosomes surface 3D reconstruction. Panel (a): the round spermatid acrosome is singled out within one of the fields of a 3D confocal stack of the experimental slide. The spermatid surface is identified thanks to the SP56 marker of its acrosomal matrix (in green). Panel (b): the Active Contour plugin reconstructs the acrosome mesh by furnishing the closest three dimensional segmented surface to the acrosome bilipidic membrane. For a 3D rendering of the acrosome mesh see Supplementary Video S1. Panel (c): acrosome mesh and the local Gaussian curvature superimposed on each mesh node. The color code is from blue (low Gaussian curvature) to red (high Gaussian curvature). Panel (d): acrosome mesh and the local Mean curvature superimposed on each mesh node. The color code is from blue (low Mean curvature) to red (high Mean curvature). Panel (e): the spermatozoon acrosome is singled out within the confocal stack field, and identified thanks to the SP56 marker of its acrosomal matrix (in green). Panel (f): the Active Contour plugin reconstructs the acrosome mesh by furnishing the closest three dimensional segmented surface to the acrosome bilipidic membrane. Notice the typical harpin shape. For a 3D rendering of the acrosome mesh see Supplementary Video S2. Panel (g): acrosome mesh and the local Gaussian curvature superimposed on each mesh node. Color code is as in panel (c). Panel (h): acrosome mesh and the local Mean curvature superimposed on each mesh node. Color code is as in panel (d).

**Figure 3:**
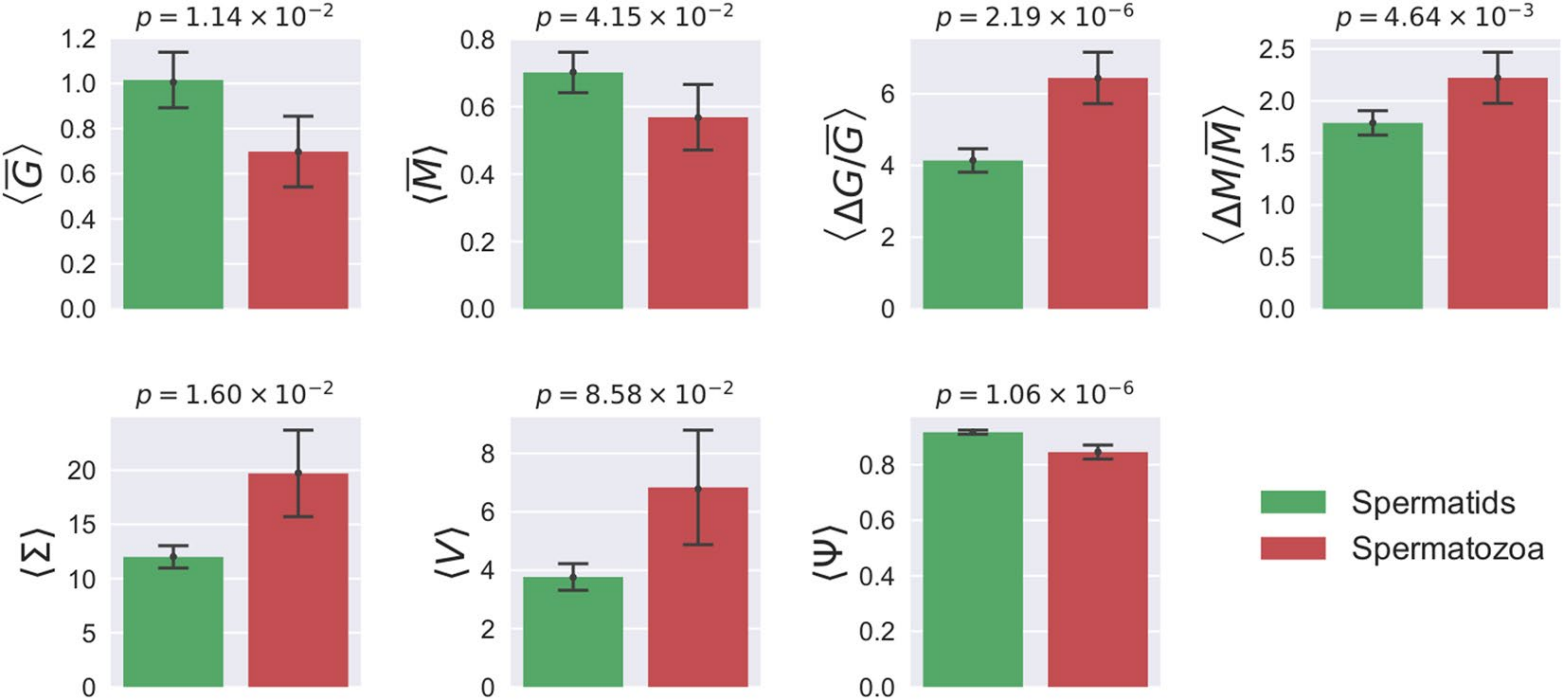
Statistical analysis: Average values. Average values of the morphological parameters for spermatids (green) and spermatozoa acrosomes (red). We also report the p-value from a KS test on top of each morphological parameter.

The detailed procedure for the reconstruction of the acrosomes surfaces, is discussed in the Materials and Methods section. Figure 2 shows two typical examples of meshes obtained by 3D reconstruction of the acrosomes membranes. The analysis of each acrosome yields a set of morphological characteristics (parameters): the acrosome’s volume *V*, its surface area Σ, the sphericity Ψ, the average mean and Gaussian curvatures ($\overline{M}$ and $\overline{G}$, respectively) and their relative fluctuations ($\frac{{\rm{\Delta }}M}{\overline{M}}$ and $\frac{{\rm{\Delta }}G}{\overline{G}}$, respectively). Averaging these morphological parameters (〈…〉) over the subpopulations of spermatids and spermatozoa gives the values reported in Fig. 3. Moreover we also report on top the *p*-values from a Kolmogorov-Smirnov test that considers the entire Spermatids and Spermatozoa cumulative distributions.

**Figure 4:**
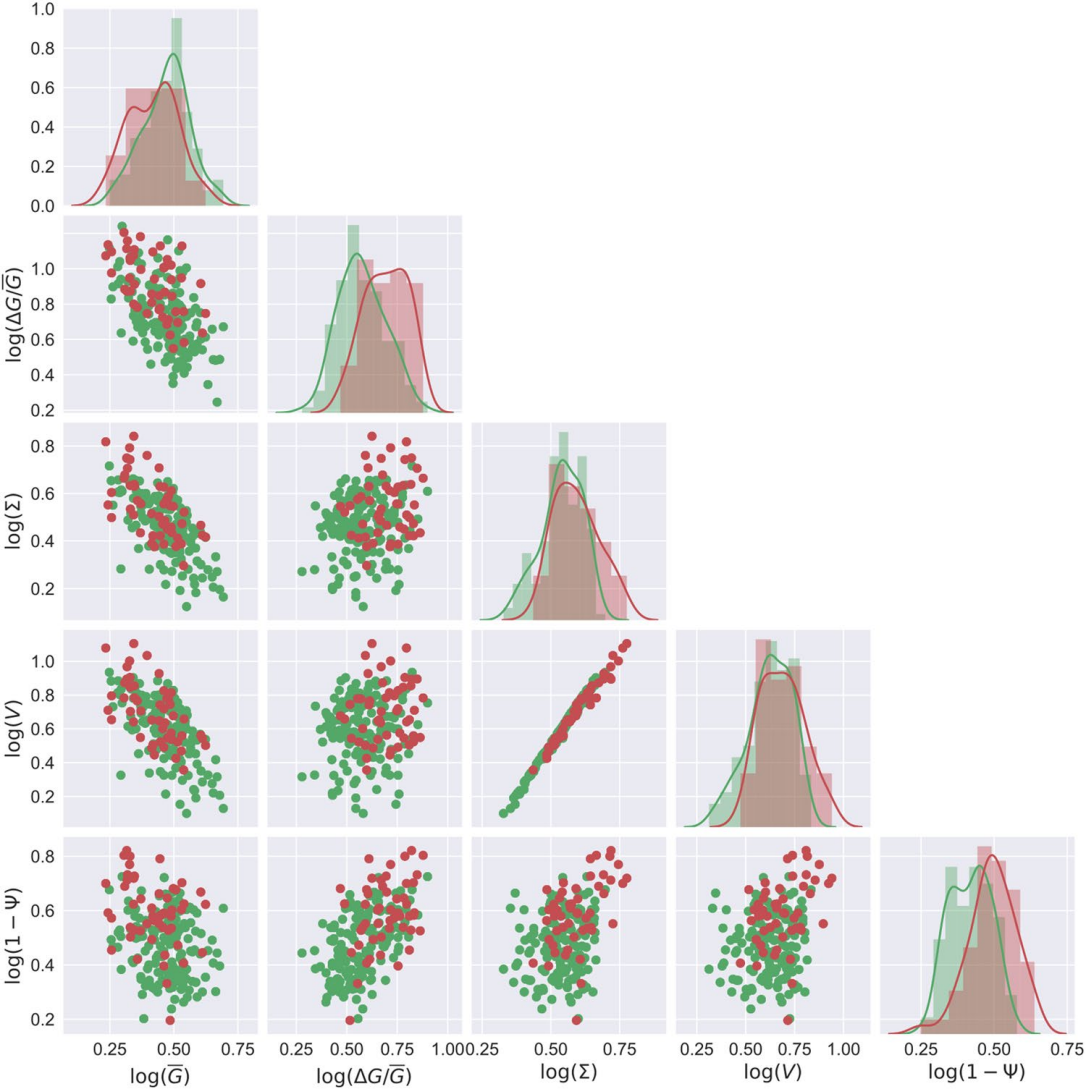
Features plot. Overall view of the distribution of five morphological features ($\overline{G}$, Δ*G*/$\overline{G}$, Σ, *V*, Ψ) and their bivariate relations. Diagonal panels: normed histograms  (semi-transparent filled bins) and kernel density estimates (solid colored lines) corresponding to the log-transformed data. Lower-diagonal panels: scatter plots in logarithmic coordinates. Notice that the x-axes are shared within columns. The diagonal panels are in units of density (not shown).

These data show that the acrosomes in spermatozoa are, in average, nearly 50% larger than those in spermatids and similar differences are recorded for the surfaces. This is not surprising since volume and surface are strongly correlated as illustrated in Fig. 4. In particular, volumes and surface follow the general law 〈Σ〉~〈*V*〉^2/3^ as expected based on simple dimensional considerations.

During spermiogenesis, acrosomes from spermatids are typically more spherical than those capping the spermatozoa nuclei. The spherical shape is probably reminiscent of an early vesicle form. We recover this observation by measuring the sphericity 1 of each acrosome in both populations. By definition, when Ψ = 1 the spherical shape is recovered, while smaller values indicate eccentricity and/or asymmetry of the surface. The mean values reported in Fig. 3 confirm indeed that acrosomes from spermatids tend to be more spherical than those from spermatozoa (see also the reconstructed meshes in Fig. 3). This difference is statistically significant (*p* = 1.06 × 10^−6^).

To further characterize the morphology, we have considered surface curvatures. The Gaussian curvature, defined in Eq. 2, is positive for spheres, negative for hyperboloids and zero for planes. Hence, the sign of the Gaussian curvature indicates if a surface is locally convex or saddle-like. We have measured the average Gaussian curvature $\overline{G}$ per cell, as defined in Eq. 5. The average value 〈$\overline{G}$〉 clearly shows that spermatids tend to have a more convex acrosome membrane as compared to spermatozoa (see Fig. 3, *p* = 1.14 × 10^−2^). The mean curvature, defined in Eq. 3, is zero for a plane, constant for a sphere and, more generally, it is positive for convex surfaces and negative for concave ones. Fig. 3 shows that, as in the case of Gaussian curvature, acrosomes from spermatids appear more spherical than those from spermatozoa (*p* = 4.15 × 10^−2^). In addition to the average values of Gaussian and mean curvature, we also consider their standard deviations which display significant differences between spermatids and spermatozoa (*p* = 2.19 × 10^−6^ for the Gaussian curvature and *p* = 4.64 × 10^−3^ for the mean curvature).

In summary, the quantitative morphological analysis reveals clear, statistically significant differences between spermatids and spermatozoa. These differences, however, arise at the population level and do not necessarily translate into a successful automated classification at the individual cell level. This is clear observing the plots in Fig. 4, where we report the bivariate relations and distribution for five morphological features. Notice that while these features all give rise to significant differences in the average parameters (Fig. 3), there is an important overlap in the individual values for spermatids and spermatozoa.

To overcome these problems, we decided to investigate if machine learning and principal component analysis could be useful to provide reliable information at the single cell level and more importantly to build up a predictive semi-quantitative method. Fig. 4 shows that the data display more uniform-like densities in logarithmic space (lower-diagonal panels) rather than in the original linear space (Supplementary Fig. S1). Hence the SVM classification is performed in logarithmic space. Having more uniform densities over the feature space is desirable for SVM classification, because penalties for misclassification are weighted according to their distance to the decision boundary.

**Figure 5:**
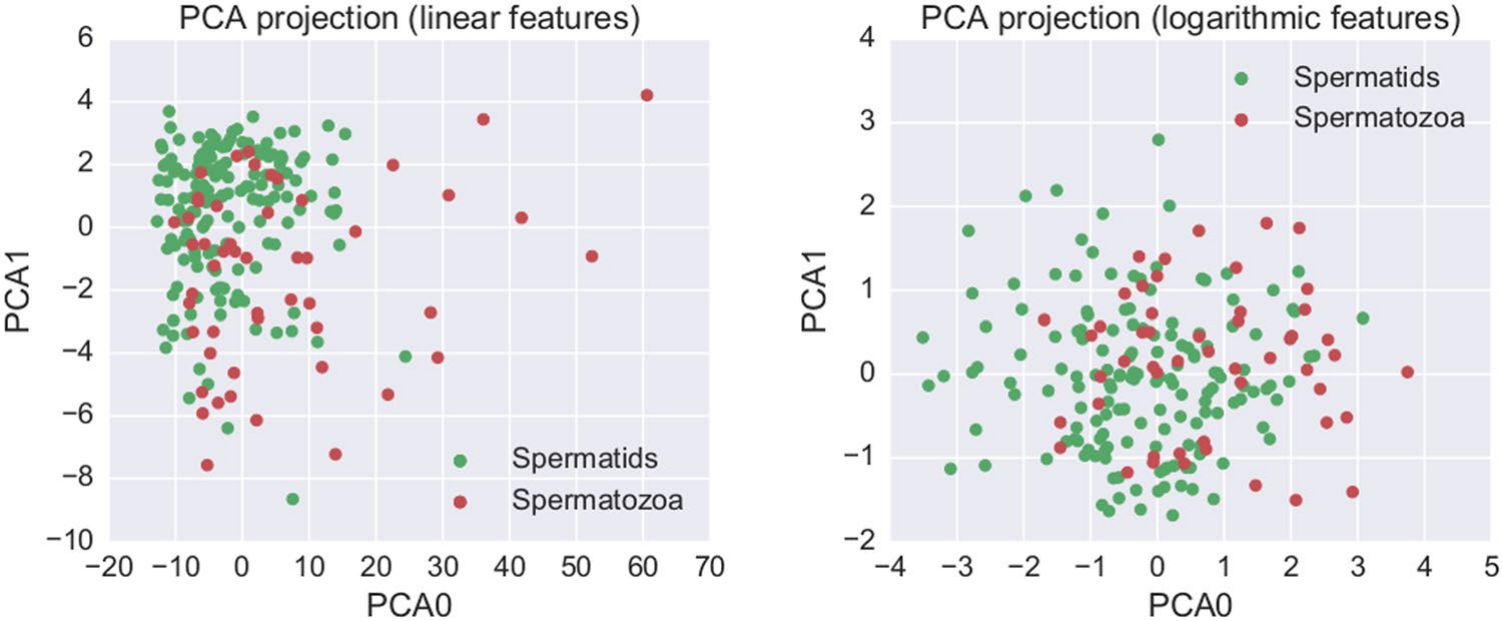
PCA projection. Projection of the seven morphological features onto its two first principal components (see Methods section), computed both in linear space (left panel) and in logarithmic space (right panel). Although some differences between spermatids and spermatozoa are apparent, no clear clusters arise.

Figure 5 shows the projection onto the first two principal components of the dataset, both in linear and logarithmic space. Although certain differences in the distribution of values for spermatids and spermatozoa can be appreciated, clearly these differences are insufficient to define non-overlapping clusters. In other words, the two subpopulations cannot be distinguished by eye in a PCA projection of the 7-feature dataset. This is, indeed, what motivated us to use a SVM in the full 7-dimensional feature space.

**Figure 6:**
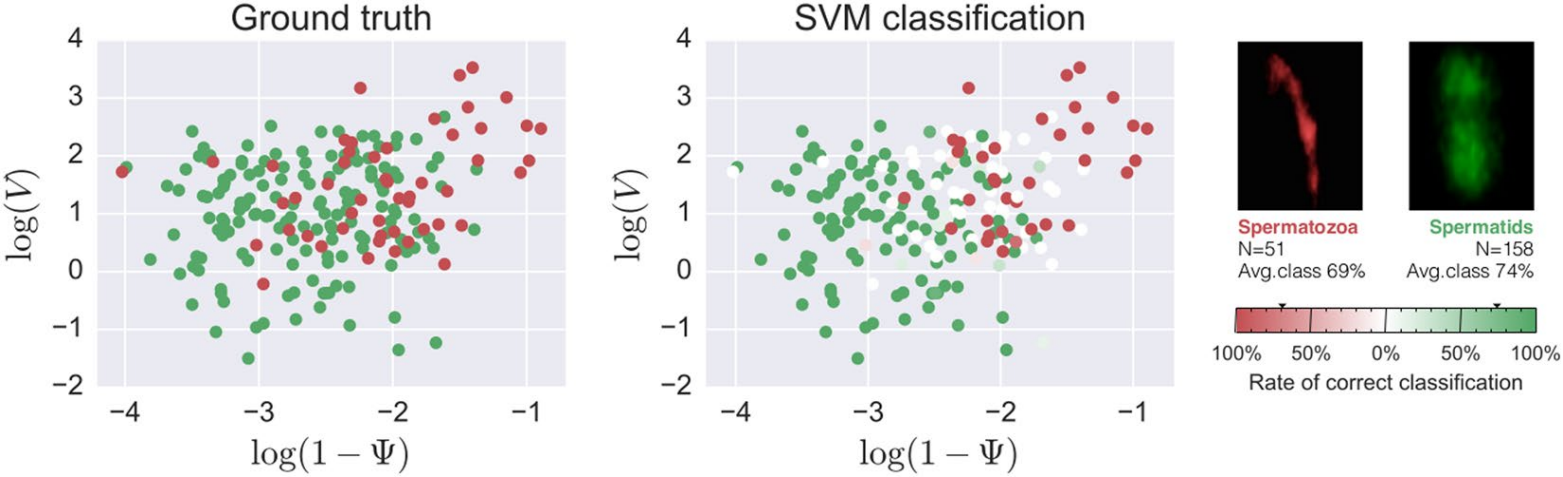
SVM analysis. Left panel: spermatids acrosomes (green dots) and spermatozoa acrosomes (red dots) plotted in the Volume-Sphericity plane. Right panel: same data, colored according to the value of the classification accuracy *A*_*c*_ (Eq. 16) obtained with the SVM: spermatids are colored from totally white (0% accuracy) to totally green (100% accuracy), while spermatozoa are colored from totally white (0% accuracy) to totally red (100% accuracy). Notice that a perfect classifier would render both panels identical. The two small images above the colorbar are example confocal images (a red coloring filter was applied to the spermatozoa image for clarity). The small triangular markers in the colorbar mark the class-level accuracy values (see Table 1).

Our results are summarized in Table 1. The values of the class accuracy (defined in Eq. 16) show that the SVM classification algorithm gets the correct answer in the 73% of trials (74% of trials for spermatids and in 69% of trials for spermatozoa acrosome, equivalent ROC AUC statistic 0.76). Although an average classification accuracy of 73% would not suffice for a potential automatized acrosome classification method, it is definitely beyond what a random or a constant classifier would achieve, marking the existing of a signal that could potentially be further exploited. In addition, it is interesting to notice the consistency by which cells are correctly classified/misclassified: 71% of all cells are correctly classified on at least 85% of the algorithm runs, i.e. *r*_*a* = 0.85_ = 0.71. If the value of *a* is raised to 0.99, then this figure drops only to 68%, i.e. *r*_*a* = 0.99_ = 0.68. In other words, there is a large subset of the data that is almost always correctly classified, and smaller subset of the data that is misclassified most of the time. This can be better seen in Fig. 6, where the cell accuracy has been used to color a scatter plot of the data. We have visually inspected the distribution of features, and found that misclassified cells lie in regions of mixed spermatid/spermatozoa density, while correctly classified ones tend to be on regions of more unequal spermatid/spermatozoa density. Therefore, it appears there is no more obvious information left, and further exploiting classification results to enhance the SVM would result in over-fitting.

**Table 1:** Summary of results of the SVM classification: class-averaged accuracy *A*_*C*_ (Eq. 16); ratio of cells with classification accuracy equal to or greater than 0.85 and 0.99, *r*_0,85_, *r*_0,99_; and area under the curve for the receiver operating characteristic (ROC AUC).

	*A*_*C*_	*r*_0.85_	*r*_0.99_	ROC AUC
Spermatids	0.74	0.72	0.70	
Spermatozoa	0.69	0.69	0.63	
All cells	0.73	0.71	0.68	0.76

The classification accuracy of each cell is defined as the ratio of times it is correctly classified, over the different runs of the algorithm (see (Eq. 17)).

The choice of SVM among other classifiers responds to its simplicity and the fact that it handles well class imbalance. In particular, we compared our result with those obtained with a Random Forest (RF) classifier using either class weights or downsampling to correct for class imbalance. In the first case, we obtain a 92% accuracy for spermatids, but only 27% for spermatozoa. In the second case, we achieve 69% accuracy for spermatids and 57% for spermatozoa. Therefore, SVM gives better results than RF, probably due to how class imbalance is handled.

In conclusion, we have proposed a general strategy to classify acrosomes from spermatides and spermatozoa according to their morphological features. The methods starts from a three dimensional reconstruction of the surface of the acrosome from confocal images and extracts a set of morphological parameters from the reconstructed surface. These parameters are then analyzed by machine learning and compared with the ground truth provided by a direct assessment by eye. The method we propose could be helpful to assist the analysis of spermatozoa during spermiogenesis, especially in presence of large quantities of data where direct classification by eye is not feasible. Future studies along these lines should aim at finding automated tools to distinguish between a normal cap and a cap with distortions of outer and inner acrosomal membranes, identify damages of the acrosomal matrix or to estimate the fraction of sperm cells with loosen cap after freezing-thawing. This could help solve the relevant clinical issue of quantifying the percentage of sperm cells with normal acrosome and therefore assess fertility.

## Methods

### Animals and culture medium

Sexually mature CD1 male mice (four-five months) were purchased from Charles River (Calco, Italy). Mice were kept in controlled conditions and all procedures were conformed to Italian law (D. Lgs n. 2014/26, implementation of the 2010/63/UE) and approved by the Animal Welfare Body of the University of Milan and by the Italian Minister of Health.

### Isolation of single cells from testis

Testes were isolated and decapsulated in 0.1 M Phosphate Buffer. The seminiferous tubules were gently placed onto a small cube made of 1,5% agarose and soaked in culture medium for more than 24 h to replace water. The amount of medium was adjusted in order to cover half to four fifth of the height of agarose cubes. Tubules were maintained in incubator at 34 °C, 5% and controlled humidity overnight in the following culture medium: RPMI (Euroclone), 10% Fetal Bovine Serum (FBS) (Euroclone), 2 mM Stable L-glutamine (Euroclone), antibiotic antimycotic solution (A5955, Sigma-Aldrich). Seminiferous tubules were picked up from agarose cubes (Sigma) and fixed in 2% paraformaldehyde dissolved in PBS pH 7.2–7.4 for 10 min. A single fixed tubule was laid down onto a slide, covered with a coverslip and a gentle pressure was applied in order to allow cells to come out from the seminiferous tubule. Slides were then frozen in liquid nitrogen for further analyses.

### Acrosome staining

The slides were rinsed with ice-cold phosphate buffered saline (PBS) 1X for 5 min at room temperature (RT), fixed with cold 100% methanol for 15 min at −20 °C then incubated with 10% goat-serum in PBS for 1 h at RT. The slides were incubated with anti-sperm Protein sp56 antibody (7C5; 1:150 Life Technologies-MA1-10866) overnight at 4 °C and then incubated with anti-mouse IgM/G/A (H + L) 488 secondary antibody (1:250 Millipore-AP501F) for 1 h at RT. The slides were mounted with Prolong Gold Antifade reagent with DAPI (Life Technologies-P36935). At least 60 stack images were acquired with Leica SP2 laser scanning confocal microscope (63X).

### Transmission electron microscopy

The seminiferous tubules were fixed in loco with 2,5% glutaraldehyde (electron microscope grade) in 0,1 M phosphate buffer (PB) pH 7.2 for 3 h at room temperature. The tubules were then mounted between two layers of 1,5% agarose (Sigma) of about 2 mm in height, which was cut into small cubes 2 × 2 × 3 mm in size and postfixed in 2% osmium tetroxide in 0,1 M PB overnight at 4 °C. The samples were dehydrated in a graded ethanol series, and embedded in epoxy resin. Semithin section (1 *μ*m) were stained with toluidine blue in borax and examined by light microscopy. Ultrathin section (70 nm) were cut using a diamond knife on a Reichert Ultracut ultramicrotome, mounted on a Cu/Rh grids (200 mesh), contrasted with uranyl acetate and lead citrate, examined and photographed with a Zeiss 902 transmission electron microscope operating at 80 kV. The exposed films were developed according to common photographic techniques, captured with an Epson V700 Photo scanner with a final resolution of 600dpi and appropriately calibrated for contrast and brightness (see Fig. 1).

### 3D acrosome reconstruction by immunofluorescence images of sp56

A 3D reconstruction of the acrosome obtained from confocal images of sperm cells stained with anti-sp56 has been done with ICY software tools (http://icy.bioimageanalysis.org/). Briefly, confocal stacks (at least 80–90 stacks) were first pre-processed to extract the individual cells images. Images were picked in diverse fields of the slide, to consider all the different stages that are present in a single portion of the tubule and not to overestimate the presence of cells in a particular stage of differentiation. A minimum of twenty cells were scored and analyzed for each slide. Two subpopulations in the seminiferous tubules were considered: round spermatids and spermatozoa. The formers represent the early stage of spermatogenesis and are identified by the presence of one or two spots of condensed heterochromatin in a spheroidal nucleus. The latters show a compact chromatin, an acrosome with hooked shape and the presence of the flagellum, according to previous paper^1, 7^. Cells were singled out by tracing a region of interest (ROI) around every acrosome in each subpolpulation. Subsequently this ROI has been cropped by using the Fast crop tool. Hence, our analysis could take advantage of single high resolution images, for any acrosome under consideration. The 3D ROI of individual acrosomes were also refined by using the HK-Means plugin (http://icy.bioimageanalysis.org/plugin/HK-Means). This method performs a N-class thresholding based on a K-Means classification of the image histogram. The acrosome membrane reconstruction has been obtained by the segmentation technique implemented in the 3D Active Contour plugin (http://icy.bioimageanalysis.org/plugin/Active_Contours)^21^. The algorithm at the basis of this plugin performs three dimensional segmentation and tracking, using a triangular mesh optimized over the original signal as a target. In Fig. 2 and in the movie M1 (see the Supplementary Informations) the 3D reconstruction of a typical spermatid and a spermatozoa acrosome are displayed. The three dimensional renderings of the meshes (in Fig. 2 and movies (Supplementary Video S1 and Supplementary Video S2) were performed thanks to Paraview (http://www.paraview.org/).

### Single cell data analysis

Once each three dimensional acrosome mesh was reconstructted, we proceeded to measure its cell volume (*V*) and surface area (Σ) using Meshlab tools (http://meshlab.sourceforge.net/). The acrosome sphericity is calculated according to the definition

1 $${\rm{\Psi }}=\frac{{\pi }^{1/3}{(6V)}^{2/3}}{{\rm{\Sigma }}}$$

The local Gaussian and Mean curvatures were calculated by a custom python code, which massively makes use of vtk libraries (http://www.vtk.org/). Typical images of a spermatid and spermatozoon acrosome mesh, with superimposed local curvatures (blue-to-red) color maps, are reported in Fig. 2(c,d) and Fig 2(g,h) for Gaussian and mean curvature respectively. We label every node on the single mesh by *i* (with 1 ≤ *i* ≤ *N*), therefore the local mean and Gaussian curvature fields on each node are denoted as *M*_*i*_ and *G*_*i*_ respectively. The local curvature of a surface entails the notion of principal curvatures, *k*_1_, *k*_2_, defined as the smallest and largest one dimensional curvatures on a point. The Gaussian curvature is defined as

2 $${G}_{i}={k}_{1}^{i}{k}_{2}^{i}$$

where the index *i* runs over the nodes of an acrosome mesh (see Fig. 2(c,g)). The mean curvature instead is defined as the average of the principal curvatures:

3 $${M}_{i}=\frac{{k}_{1}^{i}+{k}_{2}^{i}}{2},$$

and has the dimension of length ^−1^. The averaged value of the Mean and Gaussian curvature on the acrosome surface are defined as

4 $$\overline{M}=\frac{\sum _{i=1}^{N}{M}_{i}}{N}$$

and

5 $$\overline{G}=\frac{\sum _{i=1}^{N}{G}_{i}}{N}.$$

Besides the average local curvature per cell (Eqs 4 and 5 respectively), we also define relative fluctuations of the local mean curvature of individual acrosomes as

6 $$\frac{{\rm{\Delta }}M}{\overline{M}}=\sqrt{\frac{\sum _{i=1}^{N}{({M}_{i}-\overline{M})}^{2}}{(N-\mathrm{1)}{\overline{M}}^{2}}},$$

and similarly for the Gaussian curvature

7 $$\frac{{\rm{\Delta }}G}{\overline{G}}=\sqrt{\frac{\sum _{i=1}^{N}{({G}_{i}-\overline{G})}^{2}}{(N-\mathrm{1)}{\overline{G}}^{2}}}$$

### Statistical analysis

The statistical analysis is performed by averaging a set of 7 morphological parameters (*V*, Σ, Ψ, $\overline{M}$, $\overline{G}$, $\frac{{\rm{\Delta }}M}{\overline{M}}$, $\frac{{\rm{\Delta }}G}{\overline{G}}$) over the statistical ensemble of the spermatids and spermatozoa acrosomes subpopulations composed by 158 spermatids and 51 spermatozoa. The statistical significance was evaluated using Kolmogorov-Smirnov tests as implemented in the python library *scipy* (https://www.scipy.org/). Our code is available at https://github.com/ComplexityBiosystems/.

### Principal Component Analysis (PCA)

We use Principal Component Analysis (PCA)^22^ as implemented in the open-source python library *scikit-learn* (https://scikit-learn.org/stable). PCA is very popular visualization and dimensionality-reduction technique based on the singular-value decomposition of the features-samples matrix. The decomposition entails a new space of uncorrelated features, where each new feature or *principal component* is a linear combination of the original features. The principal components are of interest because (i) they are uncorrelated and (ii) they are such that the *first* principal component accounts for as much variability of the original data as possible; the *second* one accounts for as much of the remaining variability as possible, and so on. In this way, projecting the data onto the first few principal components we preserve most of the variability of the data while keeping the number of features low. In this manuscript we use PCA as a visualization technique, to discard the existence of “obvious” clusters in the dataset. By projecting the data onto the two first principal components, we obtain the 2-dimensional scatter plot that better represents the original data, in terms of explained variability.

### Support Vector Machine (SVM)

We first give a brief mathematical introduction to the algorithm behind SVM, and then discuss the implementation to our problem. SVM are a set of widely-used machine learning algorithms, highly popular for their simplicity and the fact that they yield good results in many cases. Here we use its simplest version, a SVM with a linear kernel. In essence, the algorithm boils down to finding the hyper-plane *h* parametrized by $\overrightarrow{w},b$,

8 $$h:=\{\overrightarrow{x}\in {{\mathbb{R}}}^{d}:\overrightarrow{w}\cdot \overrightarrow{x}+b=0\}$$

that better separates the data ${\overrightarrow{x}}_{i}$ into the known classes *y*_*i*_ ∈ {−1, 1}. In mathematical terms, the problem is cast into an optimization problem with constrains, which is easily solved via Lagrange multipliers. In particular, one needs to find $\overrightarrow{w},b$, *ξ*_*i*_ that minimize

9 $$\frac{1}{2}{\Vert \overrightarrow{w}\Vert }^{2}+C\sum _{i}{\xi }_{i}$$

under the constraints that

10 $${y}_{i}(\overrightarrow{w}\cdot {\overrightarrow{x}}_{i}+b)+{\xi }_{i}\ge 1\quad \quad \forall i$$

where *ξ*_*i*_ ≥ 0 are auxiliary variables that allow for misclassification (a penalty proportional to the distance to the decision boundary is set for misclassified points), and *C* sets a global weight for the misclassification penalty. We refer the reader interested in mathematical details to^22^.

The hyperplane *h* is determined using only a subset of the data, called the training set, and then the labels of the rest of the data, called test set, are predicted as follows:

11 $$y\equiv {\rm{sign}}\,(\overrightarrow{w}\cdot {\overrightarrow{x}}_{ts}+b)$$

where *y* is the predicted label of a point ${\overrightarrow{x}}_{ts}$ in the test set. There are many, more involved strategies to split the data into different sets for training and prediction. The interested reader will find good introductory material in ref. 22 and references therein.

Our data is given by the seven morphological features of the acrosomes and the acrosome subpopulation to which each cell belongs (Spermatids/Spermatozoa). That is, each cell is represented by a pair (${\overrightarrow{x}}_{i},{y}_{i}$) with ${\overrightarrow{x}}_{i}\in {{\mathbb{R}}}^{7}$ a vector containing its morphological information, and *y*_*i*_ ∈ {−1, 1} a subpopulation class label, where −1 encodes for “Spermatid” and 1 for “Spermatozoa”. We use the python implementation of Support Vector Machines provided by the machine-learning library *scikit-learn* (https://scikit-learn.org/stable). In particular, we use the function “sklearn.svm.SVC()”. Given the difference in sample size of the two groups (158 spermatids and 51 spermatozoa, see the Materials and Methods), it is important to set the keyword “class_weights” to “balanced”, which effectively sets statistical weights in the computation of the error term inversely proportional to the class observed frequencies. We use 10-fold cross validation, which means that, for each run of the algorithm, the data is randomly split into ten groups: nine are used to train the SVM, i.e. to determine the parameters $\overrightarrow{w},b$ of the hyperplane, and one is used for prediction. This is repeated ten times, one for each group, so that in the end each datapoint has received one predicted label. Given the stochasticity in splitting the data, we average results over *N*_*r*_ = 1000 runs of the algorithm. Increasing *N*_*r*_ does not improve the results.

In summary, for each run of the algorithm, the output is a predicted label “Spermatid” or “Spermatozoa” for each of the 209 acrosomes, which we then compare with the ground truth. If the predicted label corresponds to the true nature of the acrosome, we assign a binary value 1, otherwise we assign 0 if it is misclassified. Thus, we obtain a binary matrix *B*_*ij*_ of size 209 × 1000 where each row represents a cell and each column a run of the algorithm.

We define the cell accuracy *a*_*i*_ as the ratio of the times a specific cell *i* was correctly classified,

12 $${a}_{i}=\frac{1}{{N}_{r}}\sum _{j=1}^{{N}_{r}}{B}_{ij}.$$

We then define the average class accuracy *A*_*C*_ as the average of *a*_*i*_ over all cells *i* of a given class *C*, where *C* can be either spermatids or spermatozoa,

13 $${A}_{C}=\frac{1}{|C|}\sum _{i\in C}{a}_{i},$$

and |*C*| is the size of the acrosomes subpopulations, i.e. |*C*| = 158 for spermatids and |*C*| = 51 for spermatozoa. Notice that *A*_*C*_ corresponds also to the average over algorithm runs *j* = 1…*N*_*r*_ of the class accuracy,

14 $$1{A}_{C}=\frac{1}{|C|}\sum _{i\in C}{a}_{i}$$

15 $$\quad =\,\frac{1}{|C|}\sum _{i\in C}(\frac{1}{{N}_{r}}\sum _{j=1}^{{N}_{r}}{B}_{ij})$$

16 $$\quad =\,\frac{1}{{N}_{r}}\sum _{j=1}^{{N}_{r}}(\frac{1}{|C|}\sum _{i\in C}{B}_{ij}).$$

Finally we define the quantity *r*_*a*_ as the ratio of cells above certain accuracy *a* in a given class *C*, i.e.

17 $${r}_{a}=\frac{|\{i\in C,{a}_{i} > a\}|}{|C|}$$

For instance, if one takes a value of *a* = 0.99, then *r*_*a*=0.99_ would indicate the (relative) number of cells that would be correctly classified with a probability equal to or higher than 99%.

All the custom codes codes are available at https://github.com/ComplexityBiosystems/.

## Electronic supplementary material

Video S1

Video S2

Supplementary Info
