# Author Correction: Probing spermiogenesis: a digital strategy for mouse acrosome classification

**DOI:** 10.1038/s41598-018-35541-x

**Published:** 2018-11-14

**Authors:** Alessandro Taloni, Francesc Font-Clos, Luca Guidetti, Simone Milan, Miriam Ascagni, Chiara Vasco, Maria Enrica Pasini, Maria Rosa Gioria, Emilio Ciusani, Stefano Zapperi, Caterina A. M. La Porta

**Affiliations:** 1 Center for Complexity and Biosystems University of Milano, via Celoria 16, 20133 Milano, Italy; 2 Department of Physics, University of Milano, Via Celoria 16, 20133 Milano, Italy; 3 CNR-Consiglio Nazionale delle Ricerche, ISC, Via dei Taurini 19, 00185 Roma, Italy; 4 ISI Foundation, Via Chisola 5, 10126 Torino, Italy; 5 Department of Environmental Science and Policy, University of Milano, via Celoria 26, 20133 Milano, Italy; 6 Department of Biosciences University of Milano, via Celoria 26, 20133 Milano, Italy; 7 Istituto Neurologico Carlo Besta, Via Celoria, 11, 20133 Milano, Italy; 8 Department of Applied Physics, Aalto University, P.O. Box 11100, FIN-00076 Aalto, Espoo Finland; 9 CNR-Consiglio Nazionale delle Ricerche, ICMATE, Via Roberto Cozzi 53, 20125 Milano, Italy

Correction to: *Scientific Reports* 10.1038/s41598-017-03867-7, published online 16 June 2017

This Article contains an error in the Methods section under subheading ‘Animals and culture medium’.

“Sexually mature CD1 male mice (four-five months) were purchased from Charles River (Calco, Italy). Mice were kept in controlled conditions and all procedures were conformed to Italian law (D. Lgs n. 2014/26, implementation of the 2010/63/UE) and approved by the Animal Welfare Body of the University of Milan and by the Italian Minister of Health.”

should read:

“Sexually mature CD1 male mice (four-five months) were purchased from Charles River (Calco, Italy). Mice were kept in controlled conditions and all procedures were conformed to Italian law (D. Lgs 27 gennaio 1992, n. 116) and approved by the Italian Minister of Health.”

