# Supplementary material for: Probing spermiogenesis: a digital strategy for mouse acrosome classification
Source: Sci Rep. 2017 Jun 16;7:3748. doi: 10.1038/s41598-017-03867-7 (PMC5473909; doi:10.1038/s41598-017-03867-7)
Supplement: Supplementary file 3 — Supplementary Info [file 41598_2017_3867_MOESM3_ESM.pdf]

Supplementary Information for Probing spermiogenesis: a digital strategy for mouse acrosome classification

Alessandro Taloni, Francesc Font-Clos, Luca Guidetti, Simone Milan, Miriam Ascagni, Chiara Vasco, Maria Enrica Pasini, Maria Rosa Gioria, Emilio Ciusani, Stefano Zapperi, Caterina A. M. La Porta

Supplementary video captions

Video S1: 3D rendering of the surface mesh reconstruction for the acrosome of a spermatid.
Video S2: 3D rendering of the surface mesh reconstruction for the acrosome of a spermatozoon.

Supplementary Figures

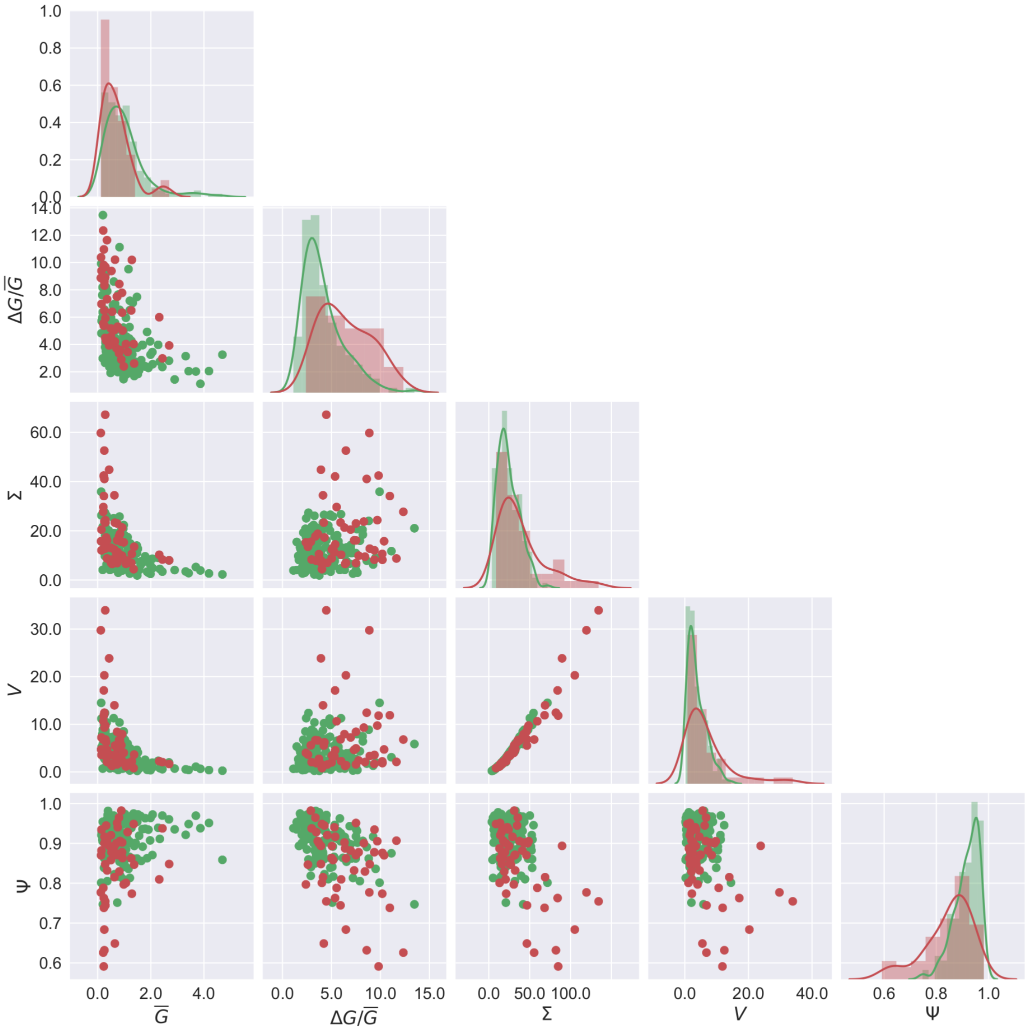

Figure S1: **Features plot.** Overall view of the distribution of five morphological features $(\bar{G}, \Delta G/\bar{G}, \Sigma, V, \Psi)$ and their bivariate relations. Diagonal panels: normed histograms (semi-transparent filled bins) and kernel density estimates (solid colored lines) corresponding to the original data in linear space. Lower-diagonal panels: scatter plots. Notice that the x-axes are shared within columns. The diagonal panels are in units of density (not shown).
